# Phosphorylation of the RNA-binding protein Zfs1 modulates sexual differentiation in fission yeast

**DOI:** 10.1242/jcs.208066

**Published:** 2017-12-15

**Authors:** Francisco J. Navarro, Probir Chakravarty, Paul Nurse

**Affiliations:** 1 Cell Cycle Laboratory, The Francis Crick Institute, London NW1 1AT, UK; 2 Bioinformatics, The Francis Crick Institute, 1 Midland Road, London NW1 1AT, UK

**Keywords:** Sexual differentiation, RNA-binding proteins, G1 cyclin, Zfs1, Puc1

## Abstract

Sexual differentiation in the fission yeast *Schizosaccharomyces pombe* promotes cell cycle arrest and extensive changes in gene expression, resulting in cell-to-cell fusion, the exchange of hereditary material and specialized cell division. These events are detrimental to the cell if they are triggered in inappropriate conditions, and therefore the decision to differentiate must be precisely controlled. Here, we investigated the role of the RNA-binding protein Zfs1 in this process by identifying its targets and characterizing novel post-translational regulatory mechanisms. We found that Zfs1 negatively regulates the G1 cyclin Puc1, and deregulated Puc1 levels inhibit differentiation in the *zfs1*Δ mutant. We also found that Zfs1 undergoes phosphorylation, which is stimulated upon nitrogen depletion or inhibition of the TOR pathway. Phosphorylation of Zfs1 modulates accumulation of Puc1 and plays an important role in the response of the cell to sexual differentiation signals. We propose that Zfs1 functions as an integrator of nutrient information to modulate sexual differentiation, contributing to the establishment of the differentiation-activating threshold.

## Abstract (precis)

**Summary:** The RNA-binding protein Zfs1 regulates accumulation of the G1 cyclin Puc1, a process that is modulated by phosphorylation. This regulatory mechanism has an important impact on cell differentiation.

## INTRODUCTION

Eukaryotic cells initiate different developmental fates in G1 phase in response to various signals including nutrient availability, growth factors and differentiation stimuli. These signals determine, for example, whether cells enter a mitotic cycle, a quiescent state, or undergo cell differentiation. Sexual differentiation in yeast is an extensively studied example of cell developmental fate (van Werven and Amon, 2011). Yeast haploid cells of different mating types arrest in the G1 phase of the cell cycle and undergo a genetic programme involving hundreds of genes that eventually leads to cell conjugation and the formation of a diploid zygote. In the fission yeast *Schizosaccharomyces pombe*, sexual differentiation is triggered by nitrogen depletion and by the exchange of pheromones between cells of different mating types (Nielsen, 2004; Yamamoto, 2004).

The decision to commit to a sexual cycle in fission yeast has important consequences for responding to environmental stress and is subject to complex regulation. At least four signaling pathways form a molecular network that controls the decision to sexually differentiate: the cAMP-signaling pathway, the stress-response mitogen-activated protein kinase (MAPK) cascade, the pheromone signaling pathway, and the TOR pathway (reviewed in Yamamoto, 2010). These pathways promote cell cycle arrest in G1 and the initiation of the genetic programme that leads to cell differentiation. Cell cycle arrest is achieved by inhibition of the cyclin-dependent kinase (CDK; called Cdc2) through upregulation of the CDK stoichiometric inhibitor Rum1, as well as by cyclin proteolysis via the anaphase-promoting complex (APC) (Stern and Nurse, 1998). The G1/S cyclins Cig2 and Puc1 play an inhibitory role in sexual differentiation (Forsburg and Nurse, 1994; Martin-Castellanos et al., 2000; Obara-Ishihara and Okayama, 1994). The specific changes in gene expression during sexual development are induced by the HMG-box transcription factor Ste11 (Sugimoto et al., 1991). Ste11 is tightly regulated by several mechanisms, including CDK-dependent phosphorylation, which restricts the DNA-binding function of Ste11 to the G1 phase of the cell cycle (Kjaerulff et al., 2007; Qin et al., 2003).

Post-transcriptional regulation is important for the control of sexual differentiation and meiosis in fission yeast, and RNA-binding proteins (RBPs) have a critical role in marking transcripts for degradation (Yamamoto, 2010). A number of RBP mutants display sexual differentiation defects (Hasan et al., 2014), with the RBPs Mei2 and Mmi1 acting as key regulators of the switch between mitosis and meiosis. Mmi1 marks meiotic-specific transcripts for degradation during the vegetative cycle, and its inactivation by Mei2-dependent nuclear sequestering is necessary for triggering meiosis (Harigaya et al., 2006). Much less is known about the role of other RBPs in the early steps of sexual differentiation. The RBPs Msa1 and Msa2 are negative regulators of sexual differentiation, probably acting on Ste11-dependent transcripts (Jeong et al., 2004; Oowatari et al., 2011), whereas the RBP Zfs1 is a positive regulator of sexual differentiation (Kanoh et al., 1995). Overexpression of *zfs1^+^* suppresses sterility caused by high levels of the adenylate cyclase Cyr1, a component of the cAMP-signaling pathway (Paul et al., 2009). Zfs1-deficient cells do not respond properly to pheromone even though Ste11 levels are not significantly altered, suggesting that Zfs1 might act on other critical regulator(s) of sexual differentiation (Kanoh et al., 1995).

Zfs1 is the only known member in *S. pombe* of a small eukaryotic family of RBPs characterized by having a highly conserved CCCH-type tandem zinc-finger domain (Blackshear, 2002; Cuthbertson et al., 2008). This domain is formed by two zinc fingers of the type CX_8_CX_5_CX_3_H separated by an 18-residue linker. Both zinc fingers are required for RNA binding, and have high affinity for adenosine and uridine (AU)-rich elements (AREs) located in the 3′ untranslated regions (UTRs) of bound mRNAs. Mutation of any of the conserved cysteine or histidine residues abrogates RNA-binding activity (Lai et al., 1999, 2002). AREs are often arranged in repeated and/or overlapping pentamers of the sequence AUUUA (Brooks and Blackshear, 2013). The most studied member of this family of RBPs is the human protein tristetraprolin (TTP, also known as ZFP36), which promotes decay of the mRNA encoding the pro-inflammatory cytokine tumor necrosis factor (TNF) and other transcripts (Brooks and Blackshear, 2013). Similar to what was found for TTP, Zfs1 promotes destabilization of transcripts containing AREs (Cuthbertson et al., 2008; Wells et al., 2012). In addition to its role in fission yeast sexual differentiation, Zfs1 is also involved in cytokinesis (Beltraminelli et al., 1999), cell size regulation (Navarro and Nurse, 2012) and cell-to-cell adhesion (Wells et al., 2012), suggesting that Zfs1 regulates a number of functionally diverse transcripts. RNA targets of Zfs1 have been identified through experiments analyzing differential gene expression in the *zfs1*Δ mutant (Cuthbertson et al., 2008; Wells et al., 2012) and mRNAs co-immunoprecipitating with Zfs1 (Hasan et al., 2014). Interestingly, this latter study showed that mating-related genes such as *gpa1*^+^, *mei2*^+^, *mfm2*^+^, *ste11*^+^ and *ste4*^+^ were downregulated in the *zfs1*Δ mutant, and that *puc1*^+^ and *ste11*^+^ mRNAs physically interacted with the Zfs1 protein. However, no further work has been done to discern the roles of these genes in the mating defect phenotype of the *zfs1*Δ mutant.

In this study, we focus on the role of Zfs1 in regulating sexual differentiation in fission yeast to obtain insights into the regulatory network that controls this developmental decision. Zfs1 immunoprecipitation (IP) and RNA-Seq data was used to identify new Zfs1 RNA targets, and we found that the G1 cyclin *puc1^+^* mRNA is negatively regulated by Zfs1. We also show that Zfs1 is phosphorylated in response to nitrogen limitation, and that Zfs1 phosphorylation modulates the sensitivity of the cell to differentiation signals. We propose a model by which Zfs1 fine-tunes the expression of *puc1^+^* to modulate the response of the cell to differentiation signals.

## RESULTS

### Identification of Zfs1 RNA targets

**Fig. 1.**
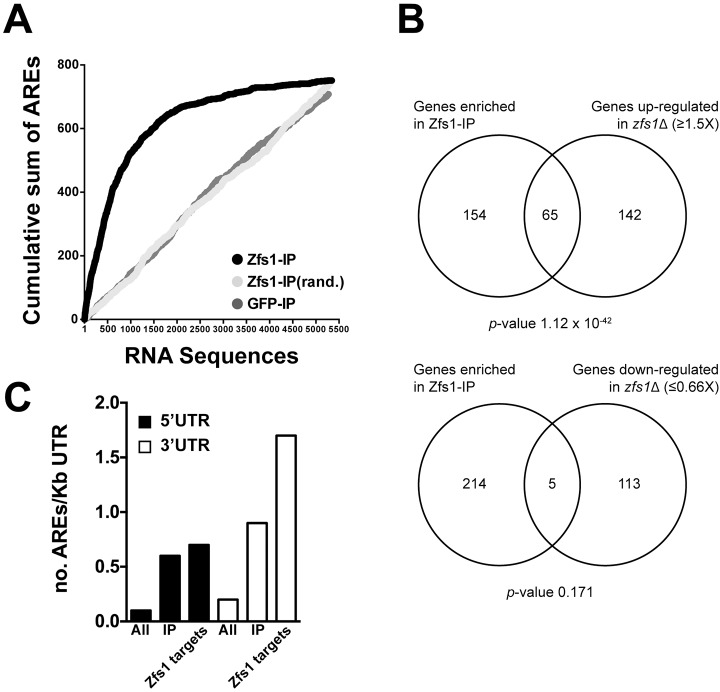
**Identification of Zfs1 RNA targets.** RNAs from WT and the *zfs1*Δ mutant strains were purified from cells growing exponentially in minimal EMM. The same growing conditions were used for cells expressing a TAP-tagged Zfs1 or a mock-control GFP construct. (A) Cumulative sum of a nonamer UUAUUUAUU AREs located on full-length transcripts. RNA sequences are ranked from higher (left) to lower (right) enrichment in the Zfs1 IP and mock-control GFP IP, or are presented in a randomized order (rand.). The number of AREs per transcript was consecutively added starting from the most enriched sequences. (B) Comparison between RNA-Seq datasets from a *zfs1*Δ mutant and Zfs1 IP-enriched sequences. Top panel, Zfs1 IP-enriched genes and upregulated genes of the *zfs1*Δ mutant. Bottom panel, Zfs1 IP-enriched genes and downregulated genes of the *zfs1*Δ mutant. *P*-values of the overlap between groups were generated using a binomial distribution (pbinom function) in R. (C) Frequency of AREs per kb of sequence in the 5′ and 3′ UTRs of the full fission yeast transcriptome (‘all’), Zfs1 IP-enriched genes (‘IP’) and Zfs1 RNA targets (‘Zfs1 RNA targets’).

The deletion mutant of the RNA-binding protein Zfs1 shows partial sterility (Kanoh et al., 1995, see also Fig. 3A), suggesting that, although cells retain the potential to differentiate, the threshold of a differentiation-activating signal is modified in this mutant. To identify RNA targets of Zfs1, we immunoprecipitated the Zfs1 protein and sequenced associated RNA molecules by using RNA-Seq (Fig. 1; Fig. S1A; Table S1). RNA-Seq was also used to identify differentially expressed transcripts in the *zfs1*Δ mutant (Fig. S1B, Table S3). The intersection of these two datasets was used to identify specific Zfs1 RNA targets. RNA immunoprecipitation (RIP) experiment replicates showed a high correlation (Fig. S1C), and did not correlate with the mock-control GFP RIP (Fig. S1D, Table S2). Zfs1 IP results also showed a high degree of overlap with the RIP data obtained by Hasan et al. (2014) (Fig. S1E). Analysis of RNAs enriched in the Zfs1 IP revealed that they contained the nonamer AU-rich-binding motif (UUAUUUAUU) at a high frequency. This motif was not enriched in a mock-control GFP IP, indicating a high specificity for our RIP assay (Fig. 1A). We selected sequences that were enriched in the Zfs1 IP experiment by more than two standard deviations from the mean (219 sequences; Table S4) and compared these with the dataset of up- or down-regulated RNAs in the *zfs1*Δ mutant (Fig. 1B). RNAs enriched in the Zfs1 IP only overlapped significantly with the upregulated (Fig. 1A, top panel) but not the downregulated gene set (Fig. 1B, bottom panel), consistent with a role for Zfs1 in destabilizing RNA. A total of 65 RNAs were simultaneously enriched in the Zfs1 IP and upregulated in the *zfs1*Δ mutant, and therefore were defined as potential *in vivo* Zfs1 RNA targets (Table S5). Of these, 64 corresponded to transcripts of protein-encoding sequences, and one to a non-coding (nc)RNA. This gene list was not enriched for any particular functional or cell component Gene Ontology (GO) category; however, the encoded transcripts had longer untranslated regions (UTRs) than the median fission yeast transcript (291 nt versus 166 nt for the 5′UTR, and 302 nt versus 257 nt for the 3′UTR), which is a feature associated with low stability (Wilhelm et al., 2008). Although AREs are normally located in the 3′UTRs of CCCH-type tandem zinc-finger RBPs targets, we found that this motif was also frequent in the 5′UTR of the IP-enriched genes and in the Zfs1 RNA target group (Fig. 1C), suggesting that Zfs1 is not only restricted to binding AREs located in the 3′UTR but also binds those in the 5′UTR. Our analysis of *in vivo* Zfs1 RNA targets therefore suggests that Zfs1 binds a set of functionally diverse transcripts.

### Zfs1 regulates *puc1*^+^ mRNA levels

**Fig. 2.**
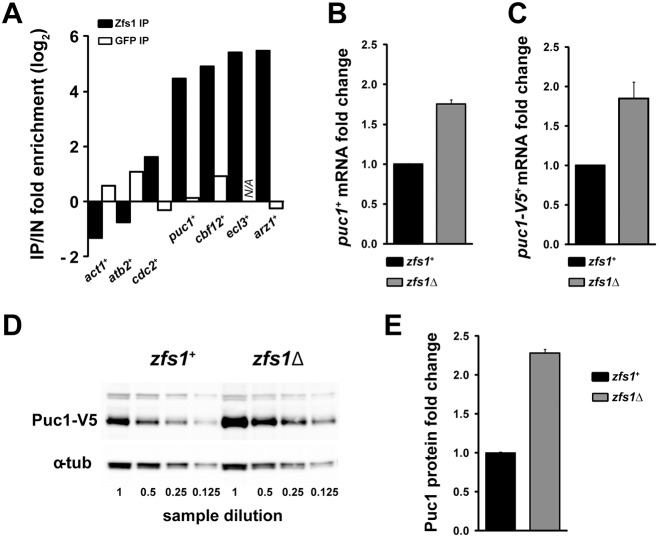
**Zfs1 regulates *puc1^+^* mRNA and protein levels.** (A) Fold enrichment of *puc1*^+^ mRNA and other transcripts in Zfs1 IP with respect to input (FDR based on *n*=3, *act1^+^* 0.035, *atb2^+^* 0.003, *cdc2*^+^ 1.46×10^−11^, *puc1*^+^ 6.01×10^−81^, *cbf12*^+^ 3.22×10^−51^, *ecl3*^+^ 1.59×10^−71^, *arz1^+^* 4.39×10^−39^). N/A, no reads for the *ecl3*^+^ transcript were detected in the GFP IP. (B) Fold change in the *puc1*^+^ mRNA levels in the *zfs1*Δ mutant. RNA was extracted from cells exponentially growing in EMM at 25°C and *puc1^+^* mRNA levels were determined by qPCR. Mean±s.e.m. from seven biological repeats are shown. (C) Fold change in mRNA levels of a V5 C-terminally tagged allele of the *puc1*^+^ gene. The V5-tagged *puc1*^+^ allele conserves endogenous UTR sequences (see Materials and Methods). Cells were treated as in B, and *puc1^+^* mRNA levels were determined by performing qPCR. Mean±s.e.m. of four biological repeats are shown. (D) Western blot showing Puc1 protein levels in the *zfs1*Δ mutant. Protein extracts were made from cells growing exponentially in EMM at 25°C. α-tubulin (Atb2) was used as loading control. (E) Quantification of Puc1 protein levels. Mean±s.e.m. from three biological repeats.

The list of potential Zfs1 RNA targets contained several cell cycle control components that have a role in sexual differentiation. We focused on the G1 cyclin Puc1 because it is one of the most enriched RNAs in the Zfs1 IP results, and it has been reported that increased levels of Puc1 inhibit sexual differentiation (Forsburg and Nurse, 1994). Puc1 was originally isolated as suppressor of the *Saccharomyces cerevisiae cln3* mutant (Forsburg and Nurse, 1991), and although it functions redundantly with other cyclins in the fission yeast mitotic cell cycle (Martin-Castellanos et al., 2000), it has been suggested that it has a role in mitotic cycle exit (Forsburg and Nurse, 1994). *puc1^+^* mRNA was specifically enriched in the Zfs1 IP to a similar level as previously characterized Zfs1 targets, such as *cbf12*^+^, *ecl3*^+^ and *arz1*^+^ (Fig. 2A). To study the effects of the *zfs1*Δ mutation on Puc1, we determined *puc1^+^* mRNA and protein levels by quantitative real-time reverse transcription PCR (qPCR) and western blotting, respectively. The RNA-Seq results for differentially expressed genes between the wild type (WT) and *zfs1*Δ mutant (Fig. S1F) were confirmed by the qPCR results, which showed that *puc1^+^* mRNA levels were 1.8 times higher in the *zfs1*Δ mutant compared to that in WT (Fig. 2B). Puc1 protein levels were assayed by using a *puc1^+^* V5-tagged allele integrated at the *puc1^+^* locus, maintaining the *puc1^+^* UTRs. Similar to what is seen with the untagged gene, the *puc1-*V5^+^ mRNA also increased in the *zfs1*Δ background (Fig. 2C), an increase that was mirrored by the protein levels (Fig. 2D,E). These results confirm that Zfs1 is a negative regulator of Puc1 levels.

### *puc1*^+^ gene deletion suppresses the sterility of the *zfs1*Δ mutant

**Fig. 3.**
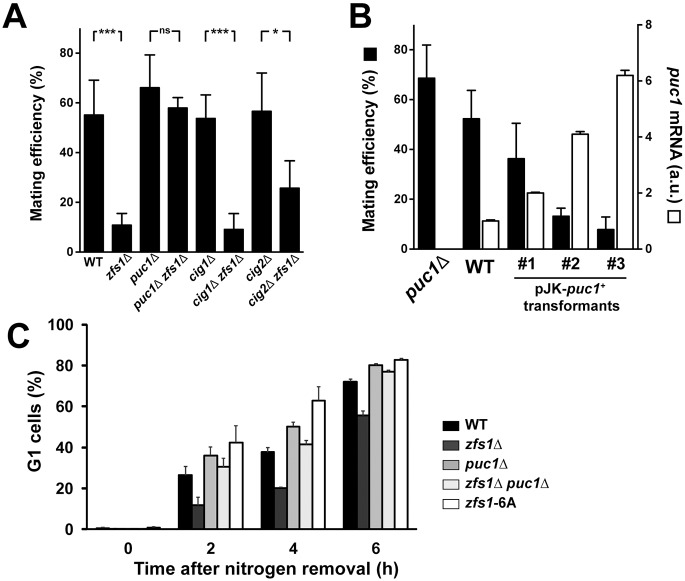
***puc1^+^* gene deletion suppresses partial sterility of the *zfs1*Δ mutant.** (A) Mating efficiency of homothallic *h^90^* strains. Cells were grown on solid YE4S medium overnight and then transferred to MEA4S plates. Plates were incubated at 25°C for 48 h before counting the number of vegetative cells and zygotes/asci. Mating efficiency was calculated as described in the Materials and Methods section. Mean±s.d. from *n*≥4, two-tailed *t*-test. (B) Dose-dependent inhibition of sexual differentiation by Puc1. *h^90^* homothallic strains carrying a different copy number of the integrative plasmid pJK148-*puc1* were assayed for their efficiency to mate. *puc1^+^* mRNA levels were measured by qPCR during vegetative growth. Mating efficiency was measured after incubation on MEA4S medium for 48 h. Mean±s.d., *n*=3. (C) Accumulation of G1-arrested cells in response to nitrogen limitation. Cells grown in minimal EMM at 25°C were washed three times with the same volume of EMM without nitrogen, and incubated in EMM without nitrogen at 25°C. At the indicated times, samples were collected and cells were ethanol fixed. The DNA content per cell was measured by propidium iodide staining and flow cytometry. Mean±s.d., *n*=3.

To assess the contribution of Puc1 to the sexual differentiation phenotype of the *zfs1*Δ mutant, we constructed a double *zfs1*Δ *puc1*Δ mutant, and assayed its mating efficiency (proportion of cells that undergo sexual differentiation, see Materials and Methods for details) as a measure of the ability of the cell to sexually differentiate (Fig. 3A). The mating efficiency of the *zfs1*Δ mutant was approximately five times lower than WT, while the deletion of *puc1^+^* gene alone did not change mating efficiency significantly. However, deletion of the *puc1^+^* gene in the *zfs1*Δ mutant increased mating efficiency to WT values, suggesting that deregulation of *puc1^+^* gene expression in the *zfs1*Δ mutant was a major contributor to the sexual differentiation defect. Puc1 has redundant functions with the Cig2 and Cig1 G1/S cyclins (Martin-Castellanos et al., 2000), therefore we assayed the specificity of the phenotypic suppression in relation to these other cyclins (Fig. 3A). Neither *cig1^+^* nor *cig2^+^* gene deletion suppressed the mating defect of the *zfs1*Δ mutant as efficiently as *puc1^+^* gene deletion, indicating a specific role of Puc1 cyclin in the phenotype of the *zfs1*Δ mutant. To test whether ectopically increasing Puc1 levels could reproduce the low mating efficiency observed in the *zfs1*Δ mutant, we integrated a variable number of plasmid copies carrying the *puc1^+^* gene into the WT strain and assayed mating efficiency (Fig. 3B). This experiment showed that Puc1 has a dose-dependent inhibitory effect over sexual differentiation, with increasing levels of Puc1 reducing mating efficiency to the levels observed in the *zfs1*Δ mutant. Furthermore, examination of the cell cycle response to nitrogen starvation, a stimulatory signal for sexual differentiation, also showed that the *puc1^+^* gene deletion could suppress defects associated with the *zfs1^+^* deletion (Fig. 3C). The *zfs1*Δ mutant was able to arrest in G1 phase upon nitrogen depletion, but it did so with slower kinetics compared with the kinetics of the WT strain, a delay in cell cycle arrest that was suppressed by the deletion of the *puc1^+^* gene. Taken together, these results indicate that Puc1 is a potent inhibitor of sexual differentiation and has a major role in the differentiation phenotype of the *zfs1*Δ mutant, highlighting the role of Zfs1 in regulating Puc1 cyclin levels for the induction of sexual differentiation.

### Nitrogen depletion and inhibition of TOR signaling promote Zfs1 phosphorylation

**Fig. 4.**
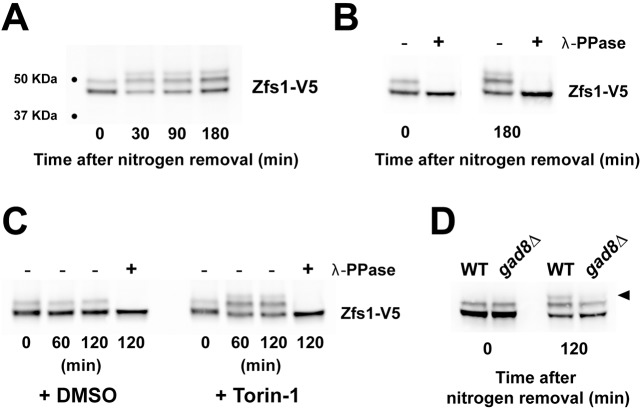
**Nitrogen depletion and inhibition of TOR signaling promote Zfs1 phosphorylation*.*** (A) Western blot of Zfs1 protein during incubation in medium without nitrogen. Cells were grown in minimal EMM to mid-log phase, then washed with medium without nitrogen and incubated in this for the indicated times. (B) Protein extracts from cells treated as in A were incubated with λ-protein phosphatase (see Materials and Methods). (C) Torin-1 inhibition of TOR complexes stimulates Zfs1 hyper-phosphorylation. Cells growing in EMM were incubated with 10 µM Torin-1 or the same volume of DMSO for the indicated times. (D) The AKT1-related Gad8 kinase is required for hyper-phosphorylation of Zfs1. Cells were grown in EMM and then washed and incubated in EMM without nitrogen for 2 h.

The function of the human homolog of Zfs1, TTP, is regulated by phosphorylation (Brooks and Blackshear, 2013). Analysis of Zfs1 by western blotting revealed slow-migrating protein species that became a fast-migrating band after phosphatase treatment, indicating that they were phosphorylated isoforms of the Zfs1 protein (Fig. 4A,B). Zfs1 migrated as two bands, corresponding to the phosphorylated and non-phosphorylated protein isoforms in cells grown in medium containing nitrogen (Fig. 4A,B, time 0 min). A short incubation in medium without nitrogen brought about the appearance of a third band corresponding to a hyper-phosphorylated isoform of the protein, indicating that Zfs1 phosphorylation responded to nutrient availability. TOR complexes are involved in the response of the cell to nutrients, and chemical inhibition of TOR activity emulates many aspects of nutrient starvation (Atkin et al., 2014). We assayed whether Zfs1 hyper-phosphorylation was regulated by TOR signaling by treating cells with the TOR inhibitor Torin-1. Addition of Torin-1 to cells growing in medium containing nitrogen was sufficient to induce the appearance of Zfs1 hyper-phosphorylation (Fig. 4C). This result suggests that Zfs1 is hyper-phosphorylated in response to the loss of TOR signaling when cells are subjected to nitrogen depletion.

To identify kinases involved in Zfs1 phosphorylation in response to nitrogen deprivation, we analyzed mutants of protein kinases that are normally upregulated in this condition and/or are required for the response of the cell to nitrogen depletion (Mata and Bähler, 2006) (Fig. S4; Fig. 4D). From this screen, we found that Gad8 kinase, which is related to human AKT-1, was required for Zfs1 hyper-phosphorylation in response to nitrogen depletion.

In summary, our results show that Zfs1 is partially phosphorylated in growth medium, and that phosphorylation of Zfs1 is stimulated by nitrogen deprivation (or loss of TOR signaling) in a Gad8-dependent manner.

### Identification of phosphorylation sites in Zfs1 protein

**Fig. 5.**
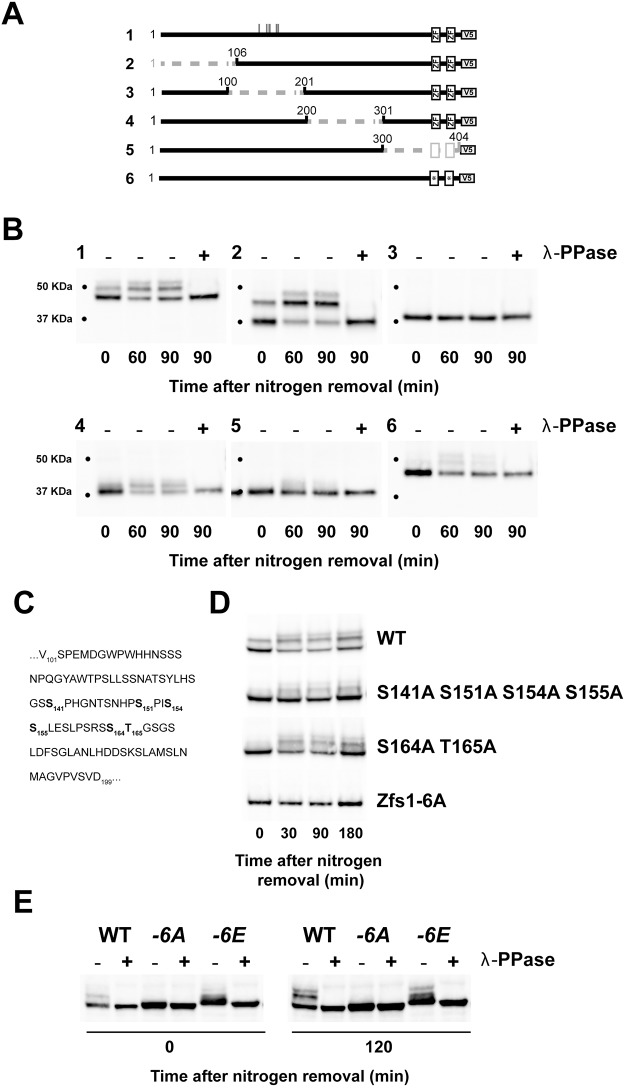
**Identification of phosphorylation sites in the Zfs1 protein.** (A) Constructs used for the identification of phosphorylation sites. Truncations of ∼100 residues were made along the Zfs1 protein and introduced at *zfs1^+^* locus to obtain WT expression levels. Boxes represent zinc finger (ZF) domains. In construct no. 6 the full-length protein harbors point mutations in two highly conserved histidine residues within the tandem zinc finger domain (H351I, H389I). Vertical lines in construct 1 mark the relative positions of residues S141, S151, S154, S155, S164 and T165. (B) Western blots to assay phosphorylation of Zfs1 constructs after nitrogen removal from the medium. Cells were treated as in Fig. 4A. (C) Amino acid sequence of the 100–201 domain of Zfs1. Residues mutated to alanine are shown in bold. (D) Western blots to assay phosphorylation of mutant Zfs1 proteins carrying the indicated mutations of serine and threonine residues to alanine residues. (E) Western blot of Zfs1 protein in mutant strains where residues S141, S151, S154, S155, S164 and T165 were mutated to alanine (-6A) or to glutamic acid (-6E).

To identify phosphorylation sites in Zfs1 protein, a series of 100-residue truncations along the protein were constructed, and phosphorylation of the protein was assayed by western blotting (Fig. 5A). Zfs1 is a 404-amino-acid protein with a tandem CCCH-type zinc finger domain near the C-terminus. Truncations of residues 1 to 105 (construct no. 2), and 201 to 300 (construct no. 4) showed robust phosphorylation both in nitrogen-containing medium and during nitrogen depletion (Fig. 5B), whereas the construct lacking the tandem zinc finger domain (truncation of residues 301 to 404, construct no. 5) exhibited a reduction in Zfs1 phosphorylated species. The reduced phosphorylation of this construct may be due to the tandem zinc finger domain being required for efficient association with the protein kinase(s), since a construct carrying point mutations in the conserved histidine residue within each zinc finger (construct no. 6) showed similarly reduced phosphorylation. Importantly, truncation of 101–200 totally abolished phosphorylation (construct no. 3) even after 90 min of nitrogen deprivation, revealing that this domain is essential for phosphorylation of Zfs1. To identify phosphorylation sites within this domain, we performed site-directed mutagenesis on serine and threonine residues, replacing them with alanine residues (Fig. 5C). Combined mutation of residues S141, S151, S154, S155 or S164 and T165 reduced phosphorylation (Fig. 5D). Moreover, the mutation of all six of these residues to alanine (Zfs1-6A) abrogated Zfs1 phosphorylation, even when cells were deprived of nitrogen for 3 h. Therefore, we conclude that these residues are important for Zfs1 phosphorylation.

Next, we constructed a strain carrying a *zfs1* allele where these same six residues were replaced with glutamic acid (Zfs1-6E) to mimic constitutive phosphorylation. In contrast to what was observed with the Zfs1-6A protein, the Zfs1-6E protein was partially phosphorylated in both nitrogen-containing and -depleted medium, as revealed by the change in mobility after protein phosphatase treatment (Fig. 5E). Taken together, these results suggest that phosphorylation of these residues is required for further phosphorylation of the protein, thus playing a key role in the regulation of Zfs1.

### Phosphorylation of Zfs1 modulates *puc1^+^* mRNA and protein accumulation

**Fig. 6.**
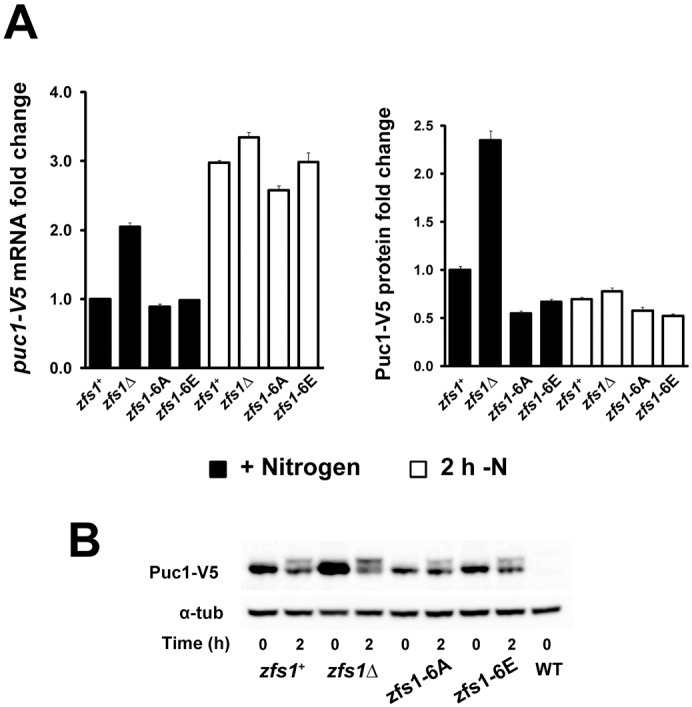
**Phosphorylation of Zfs1 regulates Puc1 protein accumulation.** (A) Relative *puc1^+^* mRNA (left panel) and protein levels (right panel) in cells growing in EMM (black bars), and after 2 h of nitrogen deprivation (white bars). Cells were washed by filtration before transferring to EMM without nitrogen. Mean values are shown; error bars correspond to s.e.m. of three technical repeats of the qPCR and from three measurements of the western blot signal. A biological replicate is shown in Fig. S6A,B. (B) Western blot of Puc1 protein levels of experiment shown in A. 0, cells growing exponentially in EMM; 2, 2 h of nitrogen deprivation. α-tubulin [α-tub (Atb2)] was used as a loading control.

We next investigated whether Zfs1 phosphorylation has a role in the regulation of Puc1 by analyzing *puc1^+^* mRNA and protein in the *zfs1* phospho-mutant strains. Untagged *zfs1* phospho-mutant strains were crossed with the *puc1-V5* strain, and samples for *puc1*^+^ mRNA and protein quantification were collected from exponentially growing cultures, and after 2 h of nitrogen deprivation (Fig. 6; a biological replicate is shown in Fig. S6A,B). In cells growing in medium containing nitrogen, *puc1*^+^ mRNA levels were similar in all strains with the exception of the *zfs1*Δ mutant, which, as has been shown above, had higher levels of *puc1*^+^ mRNA and protein than were seen in the control strain (*zfs1*^+^). Interestingly, Puc1 protein levels were reduced by approximately half in the phospho-mutant strain *zfs1-6A*, although this strain had similar mRNA levels to those found in the control strain. A strain carrying mutations of the same residues to glutamic acid to mimic phosphorylation events (*zfs1-6E*) also showed lower levels of protein than the control strain (Fig. 6; Fig. S6A,B).

*puc1*^+^ mRNA levels increased during the incubation in media without nitrogen, and reached similar levels in all strains. However, the increase in mRNA was not reflected in the Puc1 protein level, which stayed constant or decreased in the WT and *zfs1* mutant strains, indicating the action of other mechanisms during nitrogen starvation that override Zfs1 function on Puc1.

In summary, these results show that phosphorylation of Zfs1 is important for the regulation of Puc1 protein accumulation.

### Zfs1 modulates response to differentiating signals

**Fig. 7.**
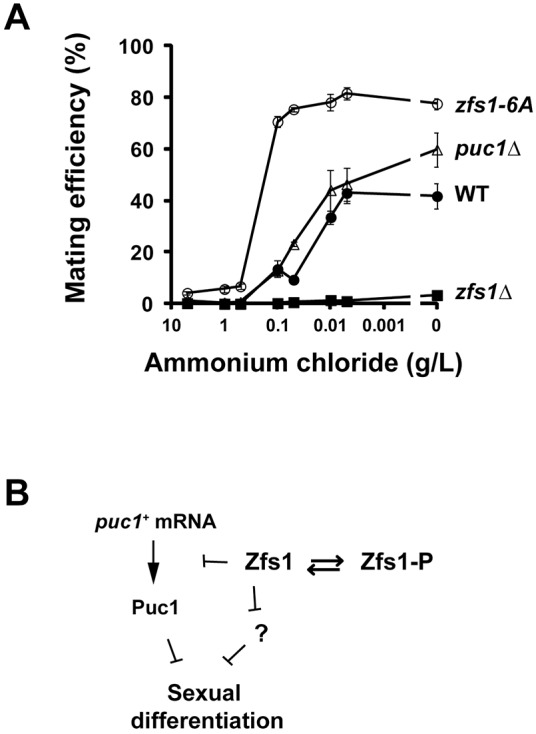
**Phosphorylation of Zfs1 modulates the response of the cell to differentiating signals.** (A) Mating efficiency at different nitrogen concentrations. Cells were grown in EMM containing 0.5 g/l ammonium chloride until they reached the exponential phase, washed twice in medium without nitrogen and diluted to a concentration of 5×10^6^ cells/ml in minimal medium containing the indicated concentration of ammonium chloride. Cells were incubated in these media at 25°C in a flat-bottom 96-well plate. Mating efficiency was determined after 24 h. Mean±s.e.m. from four biological repeats are shown (WT, *zfs1-6A* and *puc1*Δ). Mean values from two biological repeats (*zfs1Δ*) are shown. Individual experiments are shown in Fig. S7. (B) Model of Puc1 and sexual differentiation regulation by Zfs1.

To evaluate the impact of Zfs1 phosphorylation in the sexual differentiation process, we assayed the mating efficiency of the WT, *puc1*Δ, *zfs1*Δ and *zfs1-6A* strains under different nitrogen concentrations (Fig. 7A; Fig. S7). In these four strains, mating was mostly inhibited at nitrogen concentrations equal to or higher than 0.5 g/l. However, when the nitrogen concentration dropped, the induction of sexual differentiation was found to be different in each strain. In the WT strain, mating progressively increased up to 40% efficiency as the nitrogen concentration was reduced, while in the *zfs1*Δ mutant, mating was inhibited at all concentrations tested. In contrast, lowering the nitrogen concentration below 0.5 g/l in the z*fs1-6A* strain brought about an abrupt increase of mating, reaching a maximum value twice as high as in the WT strain. In agreement with the higher mating efficiency, we also observed that the *zfs1-6A* strain arrested faster in G1 (Fig. 3C). Deletion of *puc1*^+^ gene also increased mating efficiency compared to that seen in the WT strain, but to lower levels than in the z*fs1-6A* strain, in the presence of a low nitrogen concentration, reaching similar levels to that seen in the *zfs1-6A* strain in medium without nitrogen. Therefore, we conclude that Zfs1, and its regulation by phosphorylation, has an important role in modulating the cellular response to the differentiating signal brought about by falling levels of nitrogen.

## DISCUSSION

In this paper, we have studied the role of the RNA-binding protein Zfs1 in modulating the initiation of sexual differentiation. We conclude that this role is achieved in part by regulating, most likely at a post-transcriptional level, the accumulation of the G1 cyclin Puc1. We found that Zfs1 is partially phosphorylated in cells in normal growing medium, and hyper-phosphorylated when cells are deprived of nitrogen. Abrogation of phosphorylation results in changes in Puc1 accumulation and probably in other factors involved in sexual differentiation, predisposing cells to initiate sexual differentiation. It is possible that by modulating levels of Puc1 and other cell cycle/differentiation factors, Zfs1 fulfills a role in adaptation to low-nutrient conditions, maintaining cell cycle progression and preventing premature sexual differentiation (Fig. 7B).

The molecular function of Zfs1 on other RNA targets (Cuthbertson et al., 2008; Wells et al., 2012) and genome-wide decay rate measurements in the *zfs1*Δ mutant (Hasan et al., 2014), raises the possibility that Zfs1 regulates *puc1^+^* mRNA levels by promoting RNA decay. *puc1^+^* mRNA has a short half-life (Eser et al., 2016), and small changes in its turnover could be responsible for the differential expression in the *zfs1*Δ mutant. In addition, our experiments using *zfs1* phospho-mutants suggest a novel molecular function of Zfs1. The Zfs1-6A mutant had lower amounts of the Puc1 protein than the WT strain although mRNA levels were unchanged (Fig. 6), suggesting that Zfs1 may have a function in mRNA translation or protein turnover. It is possible that Zfs1 phosphorylation stimulates translation by recruiting translation initiation factors, which are rate-limiting for translation (Gebauer and Hentze, 2004), or, alternatively, that non-phosphorylated Zfs1 could have an inhibitory function over translation which is inactivated by phosphorylation. There are other examples of translational regulation of cyclins by upstream open reading frames (uORFs) (Daga and Jimenez, 1999; Polymenis and Schmidt, 1997) and by translational factors, such as DDX3/Ded1 (Grallert et al., 2000; Lai et al., 2010). Alternatively, the lower levels of Puc1 in the *zfs1-6A* strain might be the result of higher cyclin degradation. Further experiments will be required to distinguish between these possibilities. In addition to the negative effect of Zfs1, we speculate that the Puc1 protein is subject to additional downregulating mechanisms during nitrogen starvation. We observed that, even though *puc1^+^* mRNA levels were strongly increased upon nitrogen removal, Puc1 protein levels dropped in this medium (Fig. 6), a reduction which was more noticeable in the *zfs1* mutant since this strain had higher levels of Puc1 before nitrogen starvation. It is not known how the Puc1 protein is downregulated. Cig2 and Cdc13 cyclins also disappear during nitrogen starvation (Breeding et al., 1998; Wu and Russell, 1997) and, as we observed with *puc1*^+^, *cig2*^+^ mRNA levels are highly induced upon nitrogen removal (Obara-Ishihara and Okayama, 1994). The disappearance of these other cyclins seems to be a consequence of translation inhibition (Grallert et al., 2000) and/or proteolysis (Kominami et al., 1998). However, it is unclear whether these mechanisms are triggered in response to nutrient deprivation or as a consequence of cells arresting in the G1 phase of the cell cycle. The CDK stoichiometric inhibitor Rum1 is responsible for the G1 arrest during nitrogen starvation (Correa-Bordes and Nurse, 1995), and acts as a link between nutrient availability and cell cycle progression. Interestingly, the *rum1^+^* mRNA stability is regulated by nutrient availability, and removal of nitrogen from the medium stabilizes *rum1^+^* mRNA, allowing the rapid accumulation of the protein (Daga et al., 2003). It is therefore possible that nutrient signaling acts at different levels, influencing the synthesis and stability of numerous cell cycle factors.

We have shown here for the first time that Zfs1 is partially phosphorylated in actively growing cells, and that nitrogen depletion promotes further phosphorylation. Basal and nitrogen starvation-induced hyperphosphorylation of Zfs1 could have different consequences for the regulation of the Zfs1 and Puc1 protein. As mentioned above, basal phosphorylation of Zfs1 could have a role in Puc1 translation instead of mRNA stability (Fig. 6). Supporting this possible role, several reports show that phosphorylation of the human homolog of Zfs1, TTP, modulates its activity in translation (reviewed by Brooks and Blackshear, 2013). Phosphorylation is believed to facilitate translation of its targets by regulating recruitment of TTP to specific subcellular compartments or by recruiting translational factors. It is more difficult to address the effect of hyper-phosphorylation of Zfs1 during nitrogen starvation, especially on the regulation of Puc1, because of the Zfs1-independent changes in Puc1 expression that occur during nitrogen starvation (mRNA induction and reduction in protein levels). In addition to having a role in translation modulation, the phosphorylation of TPP seems to regulate other properties of the protein such as mRNA destabilization, protein stability and cellular localization (Brook et al., 2006; Marchese et al., 2010; Stoecklin et al., 2004). It is possible that the hyper-phosphorylation of Zfs1 during nitrogen starvation fulfills one of these latter functions. Further work will be required to discern the different possible roles of phosphorylation in Zfs1 regulation. We found that Gad8 is required for the hyper-phosphorylation of Zfs1 seen upon nitrogen depletion; however, we cannot rule out that the effect of this kinase on Zfs1 phosphorylation is indirect, since the *gad8* mutant cannot respond properly to nitrogen starvation nor activate the specific genetic programme for sexual differentiation (Hálová et al., 2013; Kawai et al., 2001; Matsuo et al., 2003; Weisman and Choder, 2001). Other kinases with important roles in sexual differentiation, such as Pka1 and Sty1, did not have any effect on Zfs1 phosphorylation (Fig. S4). Phosphorylation of the human homolog protein TPP is regulated by a number of signaling pathways, including the ERK MAPK, p38 MAPK, JNK, GSK3β, PKA, PKB/AKT and PKC pathways (for a review, see Brooks and Blackshear, 2013). Potential phosphorylation sites for some of these kinases are also present in Zfs1. Six residues (S141, S151, S154, S155, S164 and T165) are required for both basal and starvation-responding phosphorylation (Fig. 5), adding new phosphorylation sites to the ones identified by genome-wide proteomics (Kettenbach et al., 2015; Koch et al., 2011). Based on the behavior of the Zfs1-6E protein, we propose that phosphorylation of these residues allows further phosphorylation at other sites on the protein in response to nitrogen deprivation. However, the replacement of these residues with glutamic acid did not totally emulate phosphorylation in terms of Zfs1-mediated regulation of Puc1 levels (Fig. 6). Interestingly, mutations which impaired protein phosphorylation were found in conserved residues of the tandem zinc finger domain involved in mRNA binding, suggesting a connection between mRNA binding and phosphorylation.

We have also shown that an increase in Puc1 levels inhibits sexual differentiation. This might be due to the ability of the Puc1–Cdc2 complex to phosphorylate and probably inactivate the CDK inhibitor Rum1 (Martin-Castellanos et al., 2000). Rum1, in contrast, cannot inactivate the Puc1–Cdc2 complex, and therefore, small changes in cyclin levels can result in dramatic changes in activity. The excess of the Puc1 cyclin could extend the CDK-dependent inhibitory phosphorylation of Ste11, a master regulator of the sexual differentiation process (Kjaerulff et al., 2007). The high fertility of the *zfs1-6A* mutant, higher than that of the *puc1*Δ strain at low nitrogen concentrations (Fig. 7A), suggests that Zfs1 might regulate additional factors that prevent differentiation. Interestingly, the mRNA of the kinase Pat1 (also known as Ran1), a major regulator of sexual differentiation and meiosis (Li and McLeod, 1996; Watanabe et al., 1997), was highly enriched in the Zfs1 IP, although its RNA levels were not altered in the *zfs1*Δ mutant (Table S3). Additionally, the mRNAs of the cAMP-independent regulatory protein Pac2 (Kunitomo et al., 1995) and the heterotrimeric G protein gamma subunit Git11, a component of the cAMP/glucose-sensing pathway (Landry and Hoffman, 2001), were also identified as potential targets of Zfs1. Therefore, it is possible that Zfs1 functions as a general modulator of factors involved in sexual differentiation.

Finally, it is noteworthy that this binary regulatory mechanism formed by an RBP and a G1 cyclin that we have described in fission yeast has counterparts in budding yeast and humans (Cai and Futcher, 2013; Marderosian et al., 2006; Wang et al., 2004). In all the three organisms, an excess of G1 cyclin maintains cells in an undifferentiated state, and the RBP functions by restricting cyclin levels. In budding yeast, the G1 cyclin Cln3 is regulated by the RBP Whi3, and, although Zfs1 and Whi3 belong to different RBP families, there are parallels between the function of both proteins. Like the *zfs1* mutant, the *whi3* mutant shows low sporulation, which is rescued by deletion of the *CLN3* gene (Garí et al., 2001). In addition, the change in *CLN3* mRNA levels and translation efficiency in the *whi3* mutant is similar to the change that we described here for Puc1 in the *zfs1* mutant (Cai and Futcher, 2013). The change in Cln3 expression has been argued to be sufficient to explain the cell cycle phenotypes of the *whi3* mutant (Cai and Futcher, 2013). However, other models involving localized protein synthesis and retention have been also proposed (Wang et al., 2004). In conclusion, the regulation of G1 cyclins by RNA-binding proteins could be a conserved mechanism and provide an extra layer of regulation of cyclin levels, which can be influenced by differentiating signals.

## MATERIALS AND METHODS

### Strains and growth conditions

*S. pombe* media and methods are described in Moreno et al. (1991). Strains used are listed in Table S6. Experiments were carried out in Edinburgh minimal medium (EMM) with supplements (4S: L-histidine, L-leucine, adenine and uridine) at 0.15 mg/ml when necessary. Where indicated, YE4S (yeast extract with supplements) and MEA4S (malt extract agar with supplements) were used. When incubation without a nitrogen source was required, EMM lacking ammonium chloride was used, and cells were washed with three volumes of this medium on 0.45 µm pore Millipore filters. Torin-1 inhibitor (Tocris Bioscience) was dissolved in DMSO at 1 mM.

### Gene deletion and C-terminal tagging

Gene deletion and C-terminal tagging were performed as in Bähler et al. (1998). Gene-targeting oligonucleotides were designed using tools described on the Bähler laboratory website (www.bahlerlab.info/resources/) (Penkett et al., 2006).

### RNA extraction for RNA-Seq

RNA was extracted from a 25 ml cell culture [0.2–0.5 optical density at 595 nm (OD_595 nm_)] filtered through a 0.45 µm pore filter and the filter snap-frozen in liquid nitrogen. RNA extraction for RNA-Seq was performed with the hot phenol method as described in Lyne et al. (2003). RNA was further purified on an RNeasy mini spin column (QIAgen) following the manufacturer's instructions, and sample quality was assayed in an Agilent 2100 Bioanalyzer.

### RNA immunoprecipitation

RNA immunoprecipitation was performed as in Amorim and Mata (2009), starting from a 300 ml culture of exponentially growing cells expressing either Zfs1–TAP or mock-control GFP–TAP constructs. Cell disruption was carried out in a FastPrep cell disruptor (ThermoSavant) in Buffer A [20 mM Tris-HCl pH 8.0, 140 mM KCl, 1.8 mM MgCl_2_, Glycerol 10% (v/v), NP-40 0.1% (v/v), 10 mg/ml heparin, 1 mM PMSF and 2 µl/ml DNase Turbo (Life Technologies)] supplemented with 2× proteases inhibitor cocktail (Complete Mini-EDTA, Roche) and 30 U of recombinant RNasin (Promega). Approximately 4 mg of protein was immunoprecipitated for 2 h at 4°C with Pan mouse IgG dynabeads (Invitrogen), previously conjugated with monoclonal anti-Protein A SPA-27 antibody (cat. no. P2921, Sigma). Immunoprecipitates were washed with Buffer B [20 mM Tris-HCl pH 8.0, 140 mM KCl, 1.8 mM MgCl_2_, Glycerol 10% (v/v), NP-40 0.1% (v/v), 0.2 mg/ml heparin and 1 mM PMSF]. Finally, dynabeads were resuspended in 100 µl of lysis buffer (RNAqueous microkit, Ambion) and RNA was extracted by following the manufacturer's instructions.

### RNA sequencing and data analysis

RNA was sequenced by using 100 bp paired-end strand-specific Illumina sequencing technology. Ribosomal RNA was depleted from samples by using the Illumina Ribo-Zero Gold rRNA removal kit. All sequences were initially mapped to the *S. pombe* genome sequence, downloaded on 16 Sep 2013 from PomBase (www.pombase.org). A reference transcriptome was reconstructed by using RSEM (version 1.1.19) (Li and Dewey, 2011) from an annotation file in GTF format downloaded from PomBase. Subsequent mapping and read counting was also performed by RSEM with a seed length of 101 and a mismatch threshold of 3 in dUTP strand-specific mode. As part of the RSEM pipeline, bowtie (version 0.12.7) was used to perform the mapping stage (Langmead et al., 2009). Transcripts with count per million (CPM) value of less than 1 were eliminated. Data was normalized by using the EdgeR package, and adjusted *P*-values [accounting for the false discovery rate (FDR)] were calculated by taking into account library size and paired samples. Differentially expressed genes were also identified with the EdgeR package by using Bioconductor (version 2.7; www.bioconductor.org), running on R. Results are given as the log base 2 of the fold change of number of reads in the IP versus total RNA samples. For the final list, transcripts that had zero read counts in two of three replicate experiments were removed, as well as those with a CPM<100.

### qPCR

RNA for qPCR was extracted by using a simplified version of the hot phenol method (Schmitt et al., 1990). RNA was extracted from 10 ml cultures, and cells were incubated with phenol at 65°C for 4 min. RNA was treated with TURBO DNase before cDNA synthesis (Life Technologies). 1 µg of RNA was converted into cDNA by using random hexamer primers (SuperScript III first-strand synthesis system, Life Technologies), and qPCR was performed using EXPRESS SYBR Green ER qPCR supermix (Life Technologies). Primers are indicated in Table S7.

### Protein extraction and western blotting

Total protein extracts were prepared from 1.5×10^8^ cells. 25 ml cultures were mixed with 2.5 ml of trichloroacetic acid 100% (w/v) and incubated on ice for at least 30 min. Cells were centrifuged for 5 min at 5170 ***g***, 4°C, and the pellets washed with 10 ml of ice-cold acetone. After 5 min centrifugation at 5170 ***g*** at 4°C, cell pellets were washed twice with 500 µl of beating buffer (8 M urea, 50 mM ammonium bicarbonate and 5 mM EDTA) plus 1× protease inhibitors (Complete Mini –EDTA, Roche) and phosphatase inhibitors (PhosStop, Roche), centrifuged for 1 min at 16,249 ***g*** and resuspended in 100 µl of beating buffer plus inhibitors. Cells were disrupted with 0.5 mm glass beads in a FastPrep cell disruptor for three cycles of 35 s at 5.5 m/s, 4°C. Extracts were recovered and beads washed with an additional 50 µl of beating buffer plus inhibitors. Extracts were subjected to two consecutive centrifugations at 16,249 ***g*** for 5 min, 4°C. Finally, protein concentrations were measured by using the Quick Start Bradford reagent (Biorad). Extracts were mixed with 4× Laemmli buffer [62.5 mM Tris-HCl pH 6.8, 10% (v/v) glycerol, 1% (w/v) SDS, 0.005% (w/v) Bromophenol Blue] and heated at 72°C for 10 min before western blotting. For protein dephosphorylation assays, up to 150 µg of protein extracted with beating buffer without EDTA and phosphatase inhibitors, was treated with 400 U of λ-protein phosphatase (New England Biolabs) for 50 min at 30°C. The reaction volume was adjusted with water to dilute the urea-containing buffer at a ratio of 1:10. Protein electrophoresis was performed on 5–12% Biorad TGX gels, in a MiniProtean3 system. In western blots, V5-tagged proteins were detected with mouse anti-V5 antibody (cat. no. MCA1360, AbdSerotec, Biorad; 1:1000) and α-tubulin with monoclonal TAT1 antibody [a gift from Keith Gull, Sir William Dunn School of Pathology, University of Oxford, UK (Woods et al., 1989); 1:5000]. Horseradish peroxidase (HRP)-conjugated goat anti-mouse-IgG (GE Healthcare) was used at a dilution of 1:10,000 as secondary antibody. Signal from blots was detected by using an ImageQuant LAS 4000 (GE Healthcare) machine. Brightness was adjusted for display purposes. Uncropped original images are shown in Figs S2, S3, S5 and S6.

### Site-directed mutagenesis and allele replacement

Deletion, insertions and point mutations were created by using the Q5 site-directed mutagenesis kit (New England Biolabs) and QuikChange multi site-directed mutagenesis kit (Agilent). Point mutations and truncations of the *zfs1^+^* ORF were made on plasmid pFR145, containing the *zfs1^+^* ORF fused to the V5 tag sequence, the *ScADH1* terminator and the hygromycin resistance gene. The FR1524 (*zfs1*Δ::*ura4*^+^) strain was transformed with a fragment of the mutagenized plasmid, and *zfs1^+^* gene replacement was selected. Introduction of mutations were confirmed by sequencing. C-terminal tagging of the Zfs1 protein using the V5 peptide reduced mating significantly. Therefore, physiological experiments in the phospho-mutant strains were performed using untagged alleles, conserving the original UTRs. These alleles were constructed by introducing serine and threonine mutations on plasmid pFR157, which carries the 5′ UTR, ORF and 3′ UTR of *zfs1^+^*, plus the *leu1^+^* gene. The plasmid was integrated in the 5′ UTR of the *zfs1^+^* locus of a strain where the *zfs1^+^* ORFs were deleted by using the *ura4^+^* gene marker. 10–50 transformants were pooled and grown in YE4S medium for ∼10 generations, and 10^6^ cells were spread on 5-fluoroorotic acid (5′FOA)-containing YE4S plates containing 1 mg/ml of 5′FOA to select for cells where a recombination event between regions from the plasmid and genome has reconstituted the native *zfs1^+^* locus, eliminating plasmid sequences and the *ura4^+^* marker. *ura^−^ leu^−^* 5′FOA-resistant colonies were selected, recombination events checked by PCR, and the locus sequenced to confirm the incorporation of mutations. Strains expressing a V5 C-terminally tagged *puc1^+^* allele were constructed by transforming WT cells with a linear DNA fragment containing a partial sequence comprising the *puc1^+^* ORF, V5 coding sequencing, *puc1^+^* 3′UTR and a gene cassette conferring resistance to clonNAT antibiotic. Integration was confirmed by PCR and sequencing.

### Mating efficiency assay

Homothallic *h^90^* strains were used to determine mating efficiency. Cells were grown for 24 h on YE4S agar at 32°C and then patched on MEA4S agar at 25°C for 2 days, or grown in exponential phase in liquid EMM with low nitrogen (0.5 g/l ammonium chloride) for 36 h, washed with medium without nitrogen, and then incubated in EMM with different concentrations of nitrogen for 24 h at an initial density of 5×10^6^ cells/ml. For the mating efficiency calculation, each zygote/ascus was considered as two cells, and efficiency was calculated using the formula: 100×[2×(zygote+asci)/(2×(zygote+asci)+vegetative cells)].

### DNA content per cell determination

DNA content per cell was determined by flow cytometry as described in Navarro and Nurse (2012), using propidium iodide as the DNA stain. 10,000 cells were measured for each sample.

## Supplementary Material

Supplementary information
