# Supplementary material for: Phosphorylation of the RNA-binding protein Zfs1 modulates sexual differentiation in fission yeast
Source: J Cell Sci. 2017 Dec 15;130(24):4144–54. doi: 10.1242/jcs.208066 (PMC5769579; doi:10.1242/jcs.208066)
Supplement: Supplementary information [file joces-130-208066-s1.pdf]

Supplementary Information

Suppl Fig 1

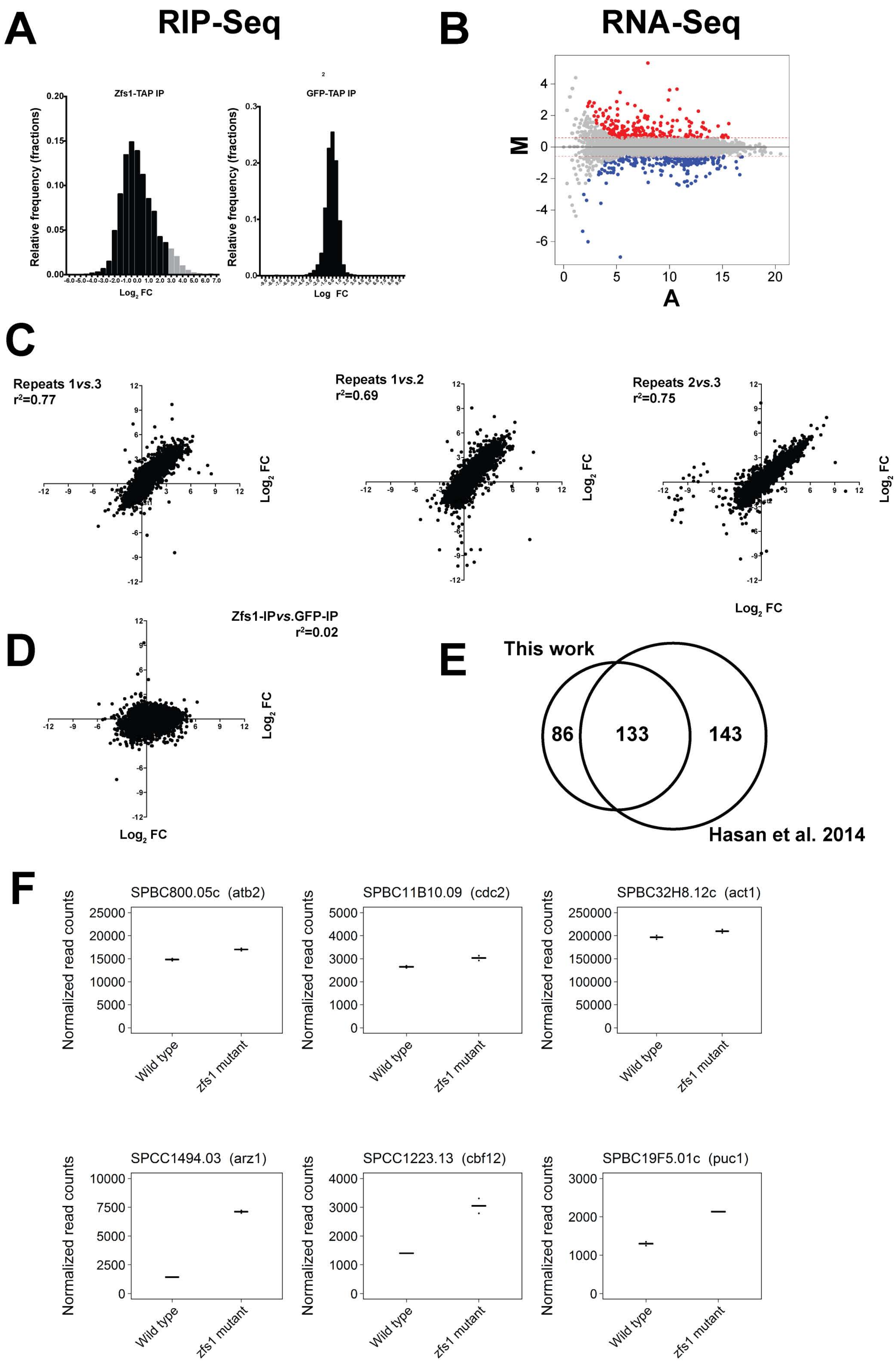

Supplementary Figure 1. Identification of Zfs1 RNA targets.

A. Distribution of sequence enrichment in the Zfs1 IP and control IP (GFP-TAP). FC, fold change enrichment of RNA sequences in the immunoprecipitate fraction with respect to input RNA. Sequences enriched in Zfs1 IP more than two standard deviations from the mean value were selected for further analysis (in grey).

B. MA plot showing differential gene expression determined by RNA-Seq between the *zfs1*Δ mutant and the wild type strain. Each dot represents a transcript, the *y* axis (M) shows log2 fold change in expression levels of the *zfs1* mutant vs. wild type, while the *x* axis (A) indicates mean RNA read counts (M=log2(zfs1/WT); A=1/2log2(zfs1xWT)). The two horizontal lines show 1.5 fold difference thresholds. Red dots mark statistically significant upregulated genes in the mutant, while blue dots show statistically significant downregulated genes (FDR <0.05). Data correspond to two biological repeats.

C. Correlation between results of three biological repeats of Zfs1 IP/RNA-Seq experiments.

D. Correlation between results of Zfs1 IP and GFP IP experiments.

E. Comparison between Zfs1 RIP results from this work and from Hasan et al. 2014 (Hasan et al., 2014).

F. Normalized read counts for selected genes in the wild type and *zfs1* mutant strains. Two biological replicates are plotted. Horizontal line shows mean of both replicates.

Journal of Cell Science • Supplementary information

Suppl Fig 2

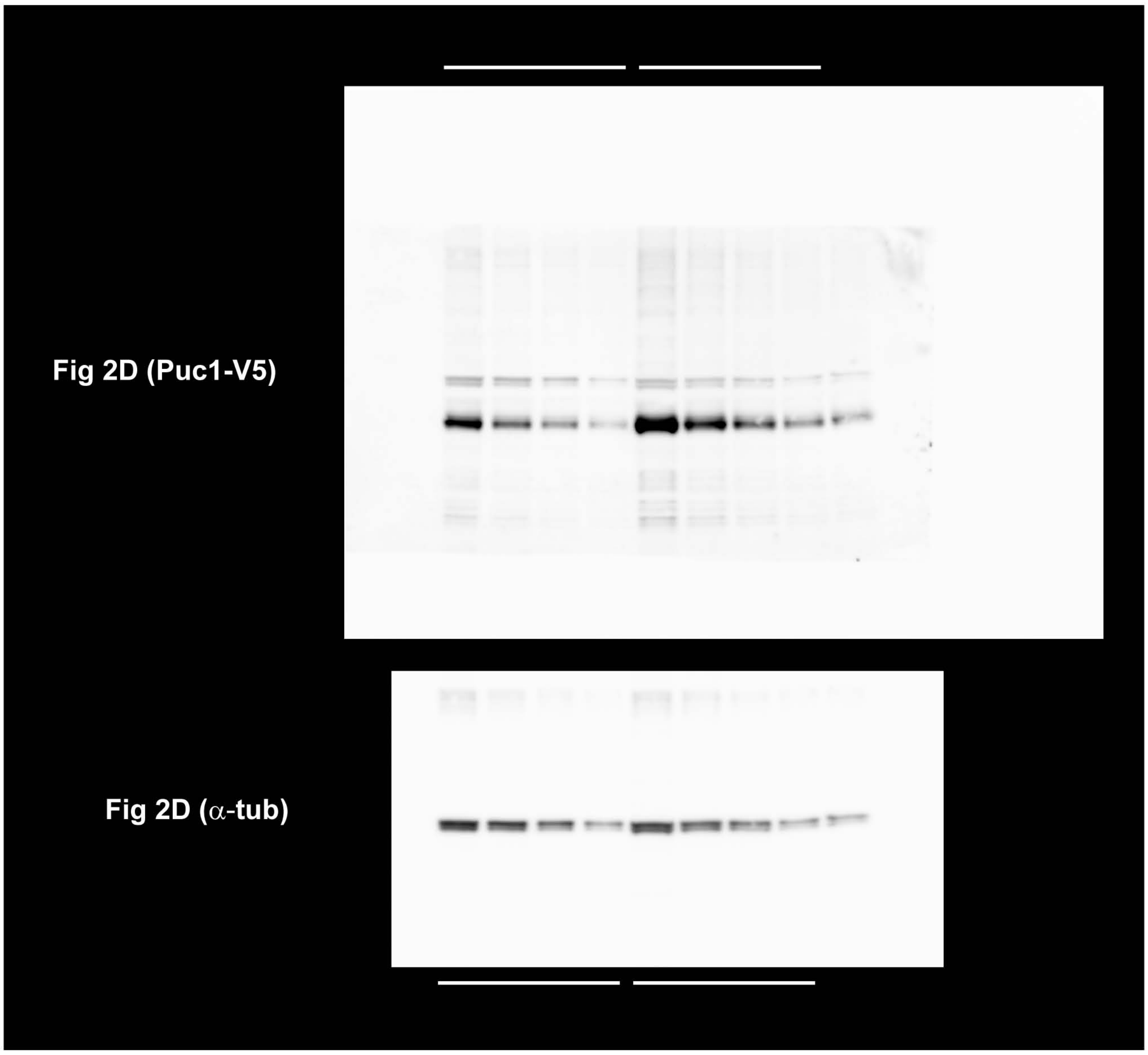

Supplementary Figure 2. Uncropped original images of Western blots used for Figure 2.

Journal of Cell Science • Supplementary information

Suppl Fig 3

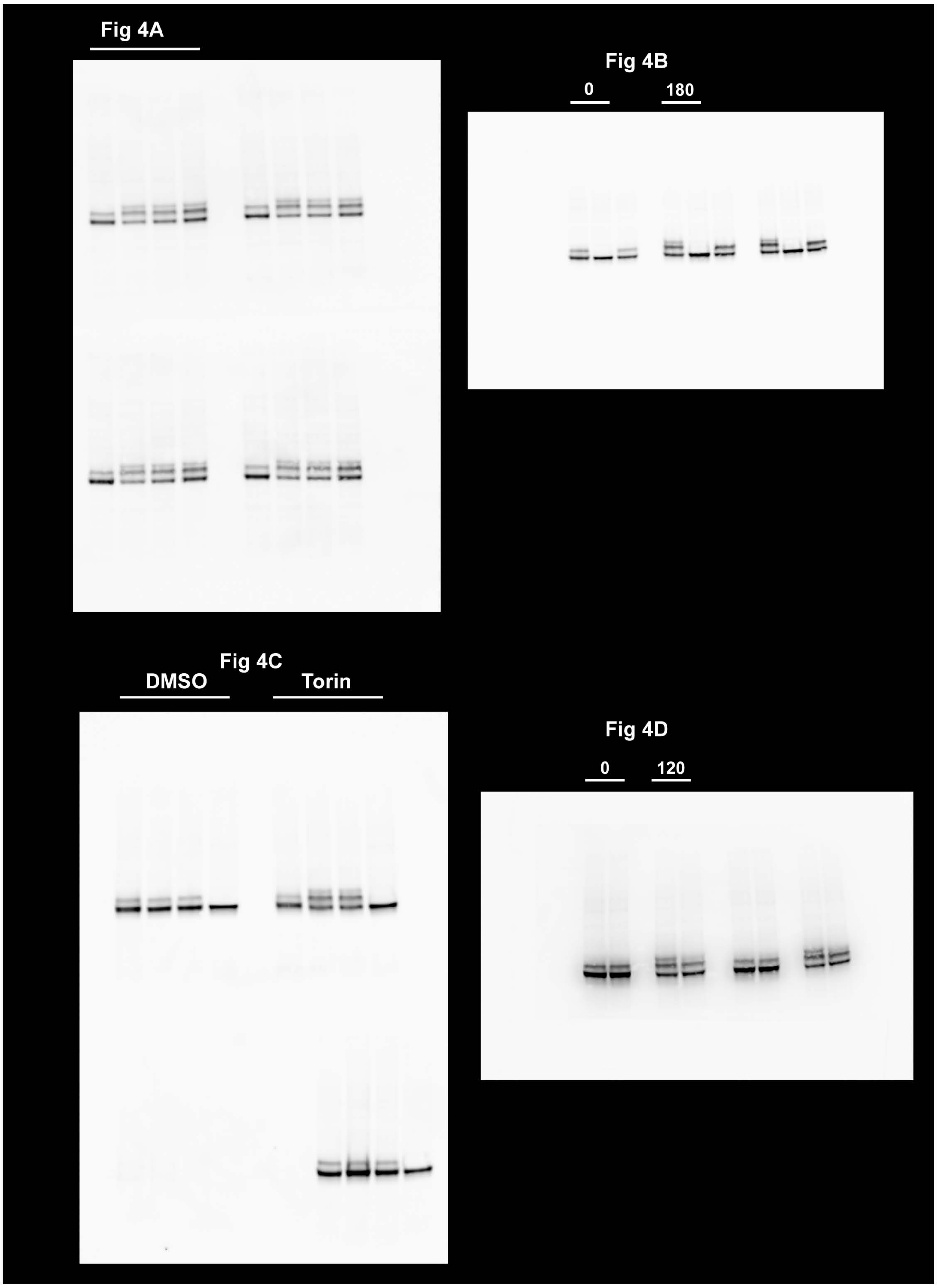

Supplementary Figure 3. Uncropped original images of Western blots used for Figure 4.

Journal of Cell Science • Supplementary information

Suppl Fig 4

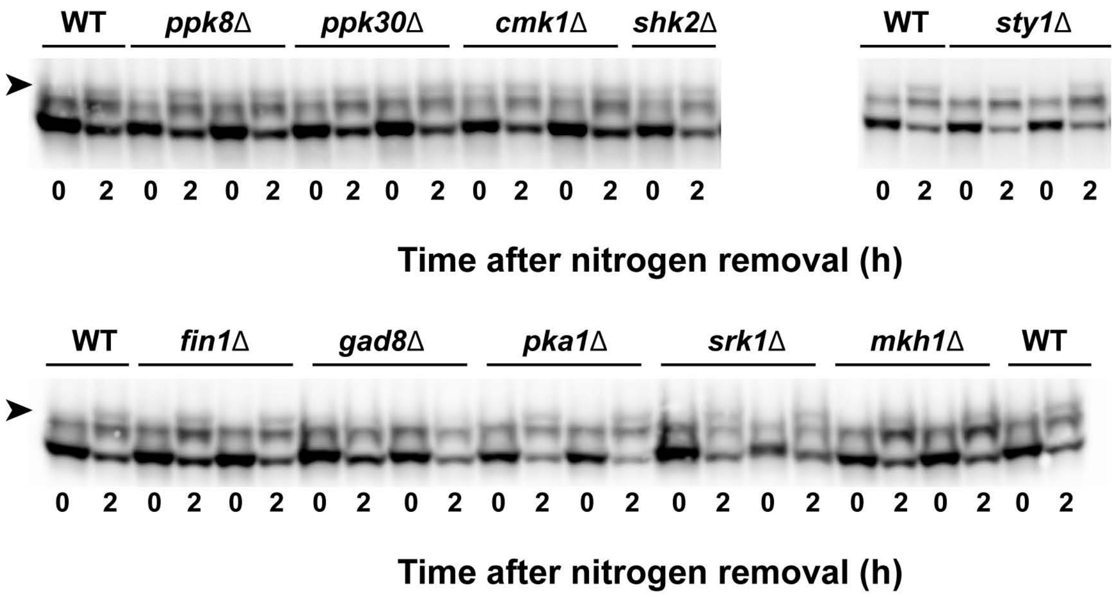

Supplementary Figure 4. Screen for kinases involved in nitrogen-regulated Zfs1 phosphorylation. Cells carrying deletions of kinase genes were grown in EMM media, washed with media without nitrogen and Zfs1 phosphorylation assayed at 0 and 120 min of incubation in nitrogen deficient media. Two biological repeats are shown for each kinase mutant. Arrow indicates the hyper-phosphorylated form of the Zfs1 protein, which is reduced in the *gad8*Δ mutant.

Journal of Cell Science • Supplementary information

Suppl Fig 5

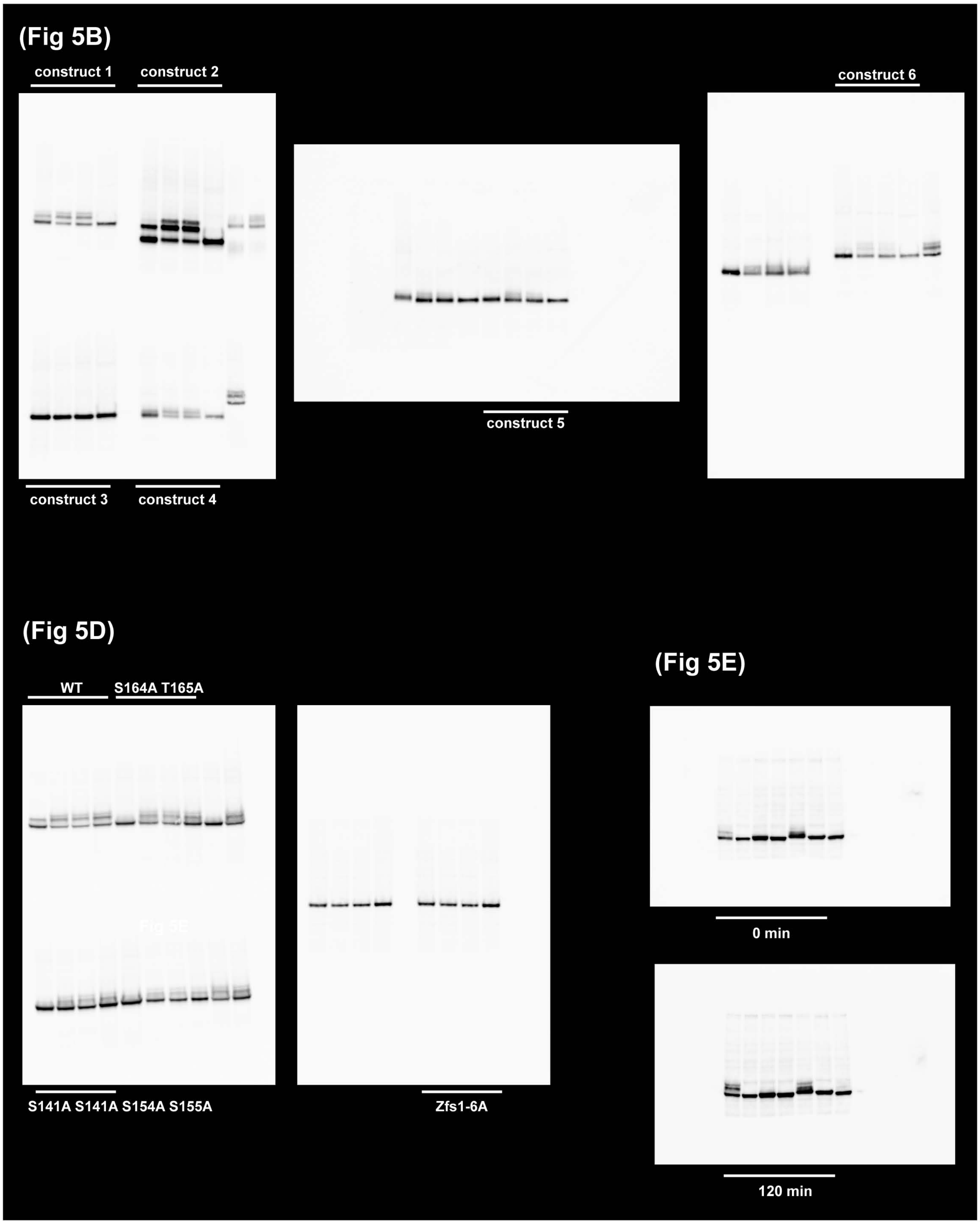

Supplementary Figure 5. Uncropped original images of Western blots used for Figure 5.

Journal of Cell Science • Supplementary information

Suppl Fig 6

A

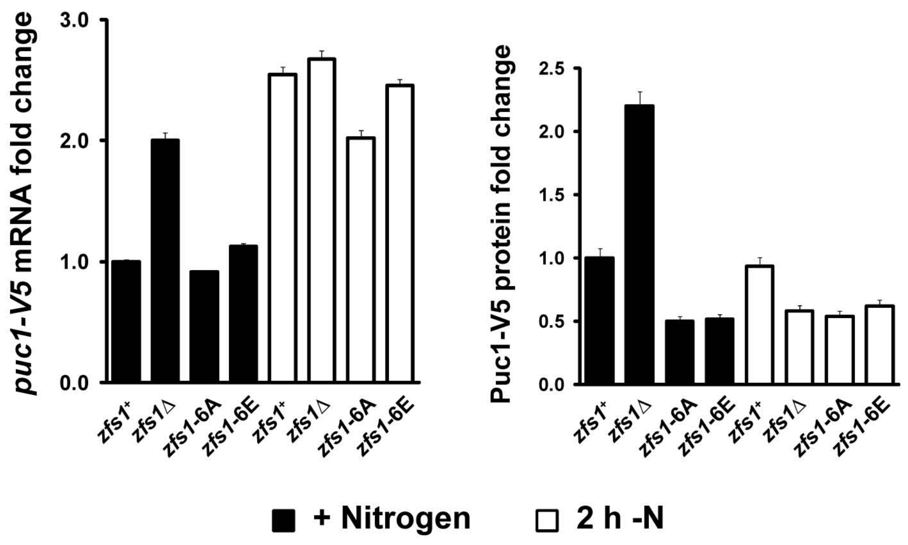

B

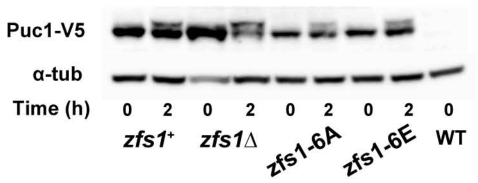

C

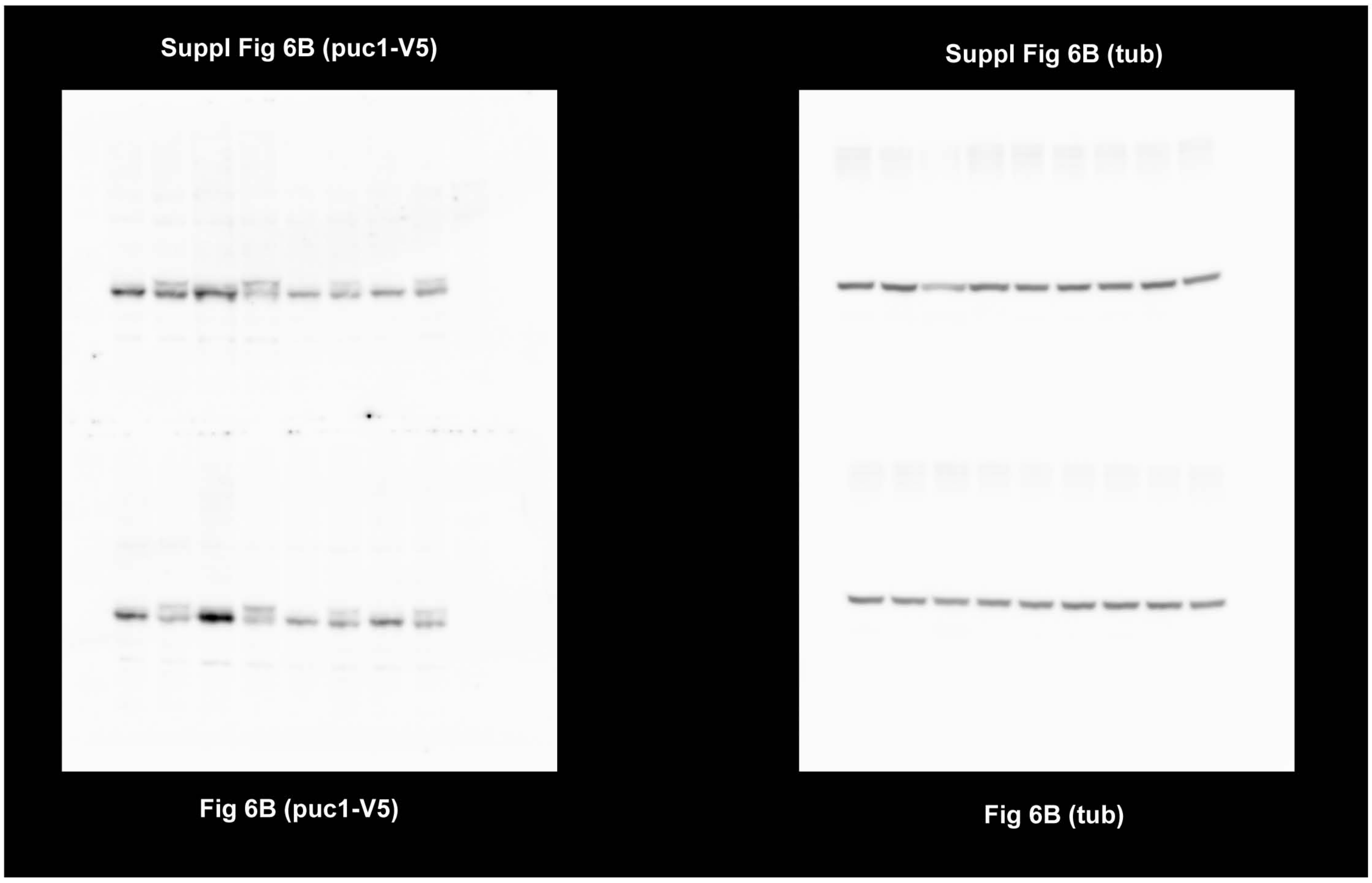

Journal of Cell Science • Supplementary information

Supplementary Figure 6. Phosphorylation of Zfs1 regulates Puc1 protein accumulation.

A. Relative *puc1*$^{+}$ mRNA (left panel) and protein levels (right panel) in cells growing in EMM (black bars), and after two hours of nitrogen deprivation (white bars). Cells were washed by filtration before transferring to EMM without nitrogen. Mean values are shown; error bars correspond to SEM of three technical repeats of the QPCR and from three measurements of the Western blot signal.

B. Western blot of Puc1 protein levels of experiment shown in A. 0, cells growing exponentially in EMM; 2, 2h of nitrogen deprivation. α-tubulin (Atb2) was used as loading control.

C. Uncropped original images of Western blots used for Fig 6B and Fig S6B.

Suppl Fig 7

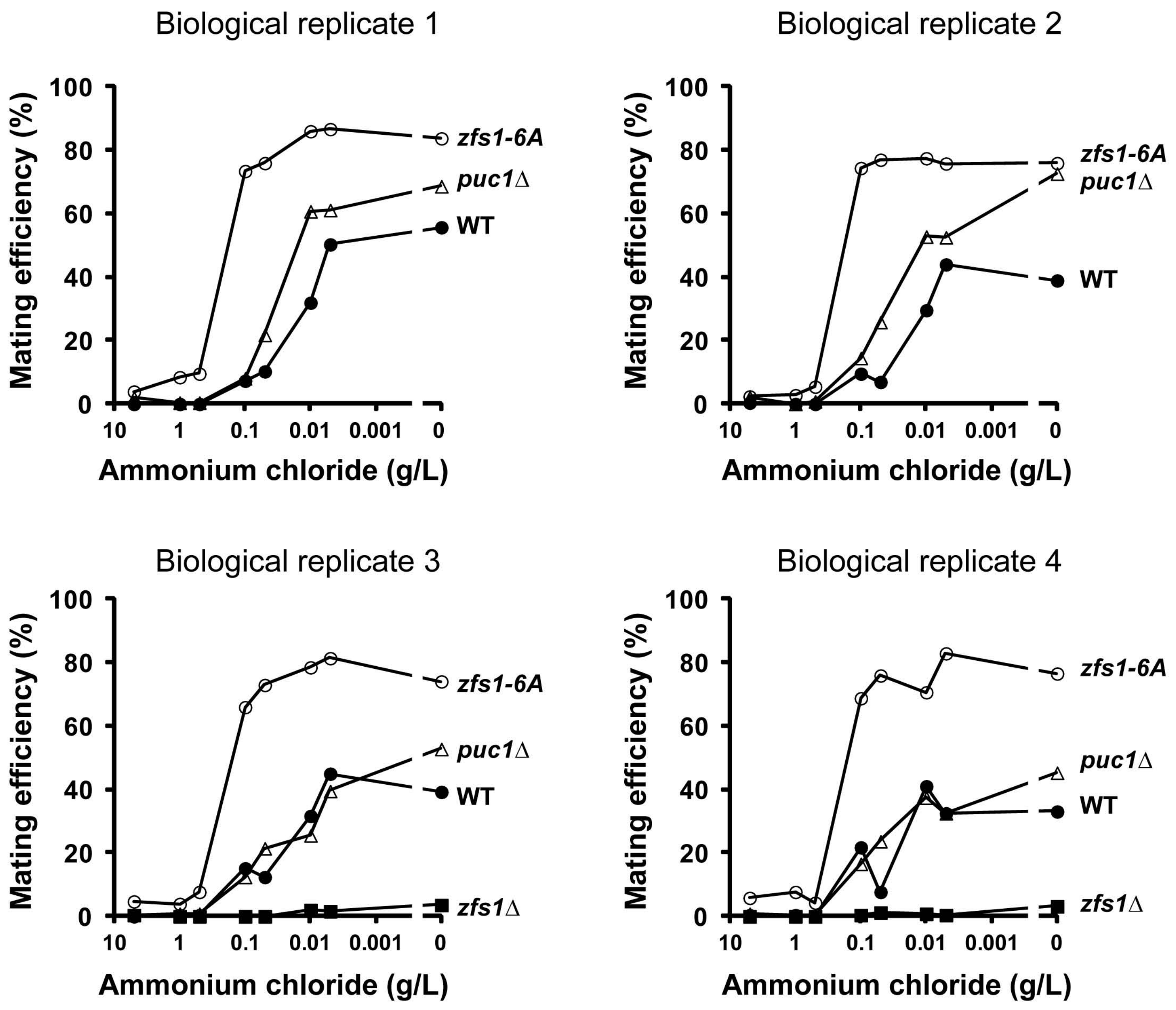

Supplementary Figure 7. Phosphorylation of Zfs1 modulates the cell's response to differentiating signals. Biological replicates of the mating assay experiment shown in Figure 7.

Journal of Cell Science • Supplementary information

Tables S1-S5

Click here to Download Tables S1 - S5

Table S6. Strains used in this work.

Name	Genotype	Origin
FR1401	*zfs1-TAP:: kanMX6 h-*	This work
FR1424	*leu1Δ::[*P_{nmt1}*-gfp-TAP-*T_{nmt1} *ura4+] ura4-D18 h+*	This work
PN2	*968* h^{90}	Lab stock
FR1519	*zfs1Δ::hphNT6* h^{90}	This work
FR1861	*puc1-V5::clonNatNT2* h^{90}	This work
FR1862	*puc1-V5::clonNatNT2 zfs1Δ::hphNT6* h^{90}	This work
FR1535	*puc1Δ::kanMX6* h^{90}	This work
FR1525	*puc1Δ::kanMX6 zfs1Δ::hphNT6* h^{90}	This work
FR1646	*cig1Δ::kanMX6* h^{90}	This work
FR1648	*cig1Δ::kanMX6 zfs1Δ::hphNT6* h^{90}	This work
FR1555	*cig2Δ::clonNatMX6* h^{90}	This work
FR1557	*cig2Δ::clonNatMX6 zfs1Δ::hphNT6* h^{90}	This work
FR1737	*puc1Δ::ura4 leu1-32 [nxpJK148-puc1] ura4-D18* h^{90}	This work
FR1738	*puc1Δ::ura4 leu1-32 [nxpJK148-puc1] ura4-D18* h^{90}	This work
FR1739	*puc1Δ::ura4 leu1-32 [nxpJK148-puc1] ura4-D18* h^{90}	This work
FR1698	*zfs1(S141A S151A S154A S155A S164A T165A)* h^{90}	This work
FR1399	*zfs1-V5:: hphMX6 h-*	This work
FR1741	*gad8Δ::clonNatNT2 zfs1-V5* h^{-}	This work
FR1502	*zfs1(Δ1-105)-V5::hphMX6 h?* (construct 2)	This work
FR1527	*zfs1(Δ101-200)-V5::hphMX6 h?* (construct 3)	This work
FR1529	*zfs1(Δ201-301)-V5::hphMX6 h?* (construct 4)	This work
FR1531	*zfs1(Δ301-404)-V5::hphMX6 h?* (construct 5)	This work
FR1479	*zfs1(H351I H389I)-V5::hphMX6 h-* (construct 6)	This work
FR1572	*zfs1(S164A T165A)-V5::hphMX6 h?*	This work
FR1574	*zfs1(S141A S151A S154A S155A)-V5::hphMX6 h?*	This work
FR1580	*zfs1(S141A S151A S154A S155A S164A T165A)-V5::hphMX6 h?*	This work
FR1839	*zfs1(S141E S151E S154E S155E S164E T165E)-V5::hphMX6 h90*	This work
FR1863	*puc1-V5::clonNatNT2 zfs1*-6A h^{90}	This work
FR1864	*puc1-V5::clonNatNT2 zfs1-6E* h^{90}	This work
FR1576	*zfs1-V5* h^{-}	This work
FR1740	*fin1Δ::clonNatNT2 zfs1-V5* h^{-}	This work
FR1743	*pka1Δ::clonNatNT2 zfs1-V5* h^{-}	This work
FR1744	*srk1Δ::clonNatNT2 zfs1-V5* h^{-}	This work
FR1742	*mkh1Δ::clonNatNT2 zfs1-V5* h^{-}	This work
FR1759	*ppk8Δ::kanMX6 zfs1-V5* h^{-}	This work
FR1760	*ppk30Δ::kanMX6 zfs1-V5* h^{-}	This work
FR1761	*cmk1Δ::kanMX6 zfs1-V5* h^{-}	This work
FR1762	*shk2Δ::kanMX6 zfs1-V5* h^{-}	This work
FR1745	*sty1Δ::kanMX6 zfs1-V5* h^{-}	This work

Journal of Cell Science • Supplementary information

Table S7. Oligos used in qPCR.

Gene	Sequence
puc1^{+}	GCTGTTTCACCAAAAGGCGTT
	AGATTCCGCTGACAATGTGCT
act1^{+}	TGTATTCCCCTCGATTGTCGG
	CACGCTTGCTTTGAGCTTCAT

Journal of Cell Science • Supplementary information
